# c-Myc-Induced Survivin Is Essential for Promoting the Notch-Dependent T Cell Differentiation from Hematopoietic Stem Cells

**DOI:** 10.3390/genes8030097

**Published:** 2017-03-06

**Authors:** Rizwanul Haque, Jianyong Song, Mohammad Haque, Fengyang Lei, Praneet Sandhu, Bing Ni, Songguo Zheng, Deyu Fang, Jin-Ming Yang, Jianxun Song

**Affiliations:** 1 Department of Microbiology and Immunology, The Pennsylvania State University College of Medicine, Hershey, PA 17033, USA; songpsulab1@gmail.com (R.H.); songpsulab2@gmail.com (M.H.); songpsulab1@yahoo.com (F.L.); songpsulab2@yahoo.com (P.S.); 2 Institutes of Irradiation/Immunology, The Third Military Medical University, Chongqing 400038, China; sdtohe@gmail.com (J.S.); nibingtmmu@yahoo.com (B.N.); 3 Department of Medicine, The Pennsylvania State University College of Medicine, Hershey, PA 17033, USA; songpsulab3@yahoo.com; 4 Department of Pathology, Northwestern University Feinberg School of Medicine, Chicago, IL 60611, USA; deyufangnwu@yahoo.com; 5 Department of Pharmacology, The Pennsylvania State University College of Medicine, Hershey, PA 17033, USA; songpsulab@gmail.com

**Keywords:** Notch signaling, c-Myc, hematopoietic stem cells, T cells, cell differentiation

## Abstract

Notch is indispensable for T cell lineage commitment, and is needed for thymocyte differentiation at early phases. During early stages of T cell development, active Notch prevents other lineage potentials including B cell lineage and myeloid cell (e.g., dendritic cell) lineage. Nevertheless, the precise intracellular signaling pathways by which Notch promotes T cell differentiation remain unclear. Here we report that the transcription factor c-Myc is a key mediator of the Notch signaling–regulated T cell differentiation. In a well-established in vitro differentiation model of T lymphocytes from hematopoietic stem cells, we showed that Notch1 and 4 directly promoted c-Myc expression; dominant-negative (DN) c-Myc inhibited early T cell differentiation. Moreover, the c-Myc expression activated by Notch signaling increased the expression of survivin, an inhibitor of apoptosis (IAP) protein. We further demonstrated that over-expression of c-Myc increased the abundance of survivin and the T cell differentiation thereof, whereas dn c-Myc reduced survivin levels and concomitantly retarded the differentiation. The c-Myc–dependent survivin induction is functionally germane, because Notch-dependent T cell differentiation was canceled by the depletion of survivin. These results identify both c-Myc and survivin as important mediators of the Notch signaling–regulated differentiation of T lymphocytes from hematopoietic stem cells.

## 1. Introduction

T cell development occurs in the thymus where the thymic microenvironment directs the differentiation of hematopoietic stem cells (HSCs). T cell precursors move to the subcapsular cortical zones of the thymus, where they encounter systems of cortical epithelial cells (the thymic stroma), and then undergo proliferation. When differentiating, these cells travel from the cortex to the medulla. The majority of HSCs that arrive in the thymus die through apoptosis without effectively accomplishing the phases needed for developing into mature naive T cells. The importance of Notch signals in these processes has been appreciated, yet the molecular mechanism underlying the Notch-regulated development of T cells from HSCs, i.e., HSC-T cells, remains elusive.

The Notch family is comprised of four Notch proteins (Notch1, 2, 3, 4) which differ in the number of epidermal growth factor (EGF)-like repeats and the existence of a transactivation domain. Mammals have five classic Notch ligands: delta-like (DLL)-1/3/4, and jagged (Jag)-1/2, which vary in the number of EGF-like repeats and the existence of a cysteine-rich domain. The engagement of the Notch receptor may result in an intracellular cleavage by *γ*-secretase that releases the Notch intracellular domain (i.e., NICD) into the cytosol. Upon nuclear translocation, NICD binds the transcriptional repressor *RBP-Jκ* and *CSL* [1,2]. NICD binding switches *RBP-Jκ* from a transcriptional repressor to an activator, subsequently initiating transcription of a number of genes. Although Notch1 receptor (N1R) is the central Notch receptor involved in T cell lineage commitment and thymic T cell maturation, the physiological ligands of N1R in these processes are not clear. The thymic epithelial microenvironment expresses all *Notch* ligands, except DLL3 which is undetectable on thymic epithelial cells (TECs) [3], and most likely not an activating ligand but a negative regulator of Notch activation [4]. Neither jagged ligand plays an essential role, as *jagged1^−/−^* and *jagged2^−/−^* mice have typical T cell development [5], indicating DLL1 and/or DLL4 ligands which support both T cell differentiation in vitro and in vivo [6]. Remarkably, conditional inactivation of DLL1 in thymocytes and/or TECs was unable to prevent T cell development [7], while inactivation of DLL4 in TECs led to a complete block in developing T cells, suggesting that DLL4 contributes a critical function throughout T cell development in the thymus [8]. Nevertheless, we have generated a different OP9 stromal cell line (i.e., OP9-DLL1/DLL4) expressing DLL1 and DLL4 molecules, and this cell line substantially induces HSCs towards CD8^+^ T lymphocyte differentiation in vitro.

In the present study, which utilized an in vitro T cell differentiation system of OP9-DLL1/DLL4, we identified the transcriptional factor c-Myc and the inhibitor of apoptosis (IAP) protein, survivin, as critical mediators of Notch signaling–regulated T cell differentiation. We show that over-expression of c-Myc increased whereas dominant-negative (DN) c-Myc reduced survivin expression, which corresponded to increased or reduced T cell differentiation. Our study demonstrates the functional role of the Notch–c-Myc–survivin axis in promoting HSC-T cell differentiation.

## 2. Materials and Methods

### 2.1. Cells and Mice

OP9 cells overexpressing DLL1 and DLL4 ligands (OP9-DLL1/DLL4) were generated by retrovirus-mediated gene introduction and enriched by fluorescent activated cell sorting (FACS). OT-I TCR-transgenic mice were bred on a C57BL/6 background and express a T-cell receptor (TCR) composed of variable (Vβ5 and Vα2) chains responsive to an ovalbumin (OVA) _257–264_ peptide (i.e., SIINFEKL). OT-I TCR transgenic and C57BL/6 mice (four- to six-week-old) were purchased from the Jackson Laboratory (Bar Harbor, ME, USA). Lck-survivin^flox/flox^ mice were kindly provided by Dr. Tak W. Mak (Ontario Cancer Institute). All experiments were carried out in compliance with the regulations of the Animal Care Committee of The Pennsylvania State University College of Medicine (#45470 and #47002), and in accordance with guidelines by the Association for the Assessment and Accreditation of Laboratory Animal Care.

### 2.2. HSC-T Cell Differentiation

CD117^+^ HSCs from the bone marrows of OT-I TCR transgenic mice were co-cultured with SNL feeder cells [9] and transduced with the retroviral constructs that express either green fluorescent protein (GFP) only or GFP plus c-Myc. HSCs (GFP^+^) were separated using a MoFlo high performance cell sorter (Dako Cytomation, Fort Collins, CO, USA), and then co-cultured with OP9-DLL1/DLL4 cells as well as cytokines, including IL-7 and Flt3L.

### 2.3. Retroviral Transduction

Mig-c-Myc-IRES-GFP (Mig-c-Myc) was obtained from Addgene (Cambridge, MA, USA), and Mig-dn-c-Myc (Δ106–143)-IRES-GFP (Mig-dnMyc) was generated as described [10]. Construction and use of Mig-dn-MAML1 (ICN13-74) was described previously [11]. Retroviral transduction was implemented as described [9]. Expression of DsRed was confirmed by flow cytometric analysis, gating on GFP^+^ cells. The gene-transduced DsRed^+^ GFP^+^ cells were isolated using a high-speed cell sorter as mentioned above.

### 2.4. PCR-Based Array and RT-PCR

Mouse Transcription Factors RT^2^ Profiler PCR Array (Cat. #PAMM-075A) was implemented with RT^2^ SYBR Green Mastermix (Cat. #330522) from Qiagen (Germantown, MD, USA) by using an ABI StepOnePlus^TM^ Real-Time PCR System from Life Technologies (Carlsbad, CA, USA), as described previously [10].

### 2.5. Western Blot

Live HSC-derived cells from the in vitro co-cultures were recovered by gentle repetitive pipetting, and the cell lysates were prepared for Western blotting as described [12].

### 2.6. Flow Cytometric Analysis

HSCs were co-cultured with OP9-DLL1/DLL4 cells for various periods, and the surface protein expression of CD117, CD25, CD44, CD4 and CD8 was examined by flow cytometry after gating on CD8^+^ cells or other markers, such as GFP expression. The Notch1 intracellular domain (Notch1^IC^) was determined by intracellular staining of HSC-derived cells using the Intracellular Fixation & Permeabilization Buffer Set (Product #88-8824) from eBioscience (San Diego, CA, USA).

### 2.7. Antibodies

c-Myc (#9402) Ab was purchased from the Cell Signaling Technology (Beverly, MA, USA). Survivin (D-8, sc-17779) and Actin (C2, sc-8432) antibodies (Abs) were obtained from the Santa Cruz Biotech (Santa Cruz, CA, USA). PE/Cy7, PerCP, PerCP/Cy5.5, or APC conjugated TCRVβ5 (Clone MR9-4), CD117 (Clone 2B8), CD25 (Clone 3C7), CD44 (Clone IM7), CD3 (Clone 17A2), TCR (Clone H57-597), CD4 (Clone GK1.5) and CD8 (Clone 53-6.7) Abs were purchased from Biolegend (San Diego, CA, USA). Notch1^IC^ (Clone mN1A) and mouse IgG1 kappa Isotype Control were purchased from eBioscience (San Diego, CA, USA). Annexin V: PE Apoptosis Detection Kit (559763) was purchased from BD Bioscience (San Diego, CA, USA).

## 3. Results

### 3.1. Notch Signaling Induces HSC-T Cell Differentiation

**Figure 1:**
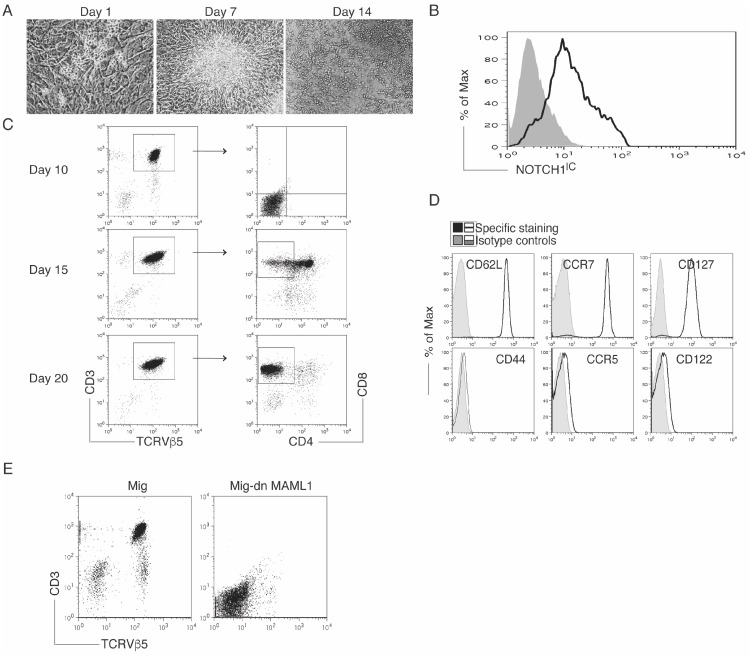
Notch signaling induces hematopoietic stem cells (HSC)-T cell differentiation. Bone marrow–derived CD117^+^ HSCs from OT-I TCR transgenic mice were either transduced with a Mig or Mig-dnMAML1, and then directly co-cultured with OP9-DLL1/DLL4 cells and cytokines (inteleukin-7 (IL-7) and FMS-like tyrosine kinase 3 ligand (Flt3L)). (**A**) Morphology of cell differentiation on days 0, 7, and 14; (**B**) On day 3, cleaved Notch1 intracellular domain (Notch1^IC^) (open histogram) was determined by intracellular staining of HSC-derived cells (filled histogram shows isotype control); (**C**) Differentiation of HSC-T cells in vitro. Flow cytometric analysis for the protein expression of HSC-derived cells on days 10, 15 and 20. CD3^+^ TCRVβ5^+^ cells were gated as indicated, and analyzed for expression of CD4 and CD8; (**D**) Phenotypic analysis of HSC-T cells. At day 20, CD3^+^ TCRβ5^+^ CD8^+^ T cells were analyzed by flow cytometry for expression of certain markers; (**E**) Dominant-negative (DN) MAML1 blocked the in vitro HSC-T cell differentiation. On day 10, live HSC-derived cells were analyzed for expression of CD3^+^ TCRVβ5^+^ cells by flow cytometry. Data are the representative of three independent experiments.

CD117^+^ HSCs from the bone marrow of OT-I TCR transgenic mice were co-cultured with OP9-DLL1/DLL4 cells and cytokines including interleukin-7 (IL-7) and FMS-like tyrosine kinase 3 ligand (Flt3L) (Figure 1A). On day 3, Notch activation was confirmed by flow cytometric analysis of Notch1^IC^ (Figure 1B). Following 10 days of co-culture, CD3^+^ TCRVβ5^+^ T progenitors were generated, and after 20 days of co-culture, OVA-specific (Vα2, Vβ5) CD8^+^ T cells differentiated (Figure 1C). OVA-specific HSC-derived CD8^+^ T cells had a naïve phenotype and greatly expressed CD62L, CCR7 and CD127, but not CD44, CCR5 and CD122 (Figure 1D).

To verify the Notch signaling–mediated HSC-T cell differentiation, CD117^+^ HSCs from the bone marrow of OT-I TCR transgenic mice were transduced with a DN Mastermind-like 1 (MAML1; required for Notch target gene activation), and then were co-cultured with OP9-DLL1/DLL4 cells and cytokines including IL-7 and Flt3L. No expression of CD3^+^ TCRVβ5^+^ cells was detected (Figure 1E). The gene transduction of the DN MAML1 substantially inhibited the in vitro early differentiation of HSC-T cells. These results clearly demonstrated the role of Notch signaling in inducing the differentiation of OVA-specific CD8^+^ T cells from HSCs.

### 3.2. Notch Signaling Regulates c-Myc Expression during Differentiation of HSC-T Cells

**Figure 2:**
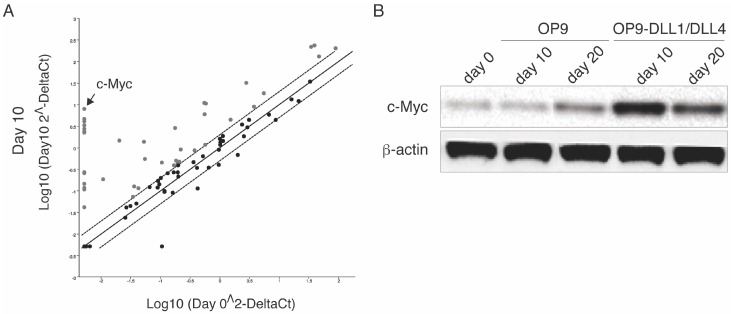
Notch signaling regulates c-Myc during HSC-T cell differentiation. Bone marrow–derived CD117^+^ HSCs from OT-I TCR transgenic mice were co-cultured with OP9-DLL1/DLL4 or OP9 cells. HSC-derived cells were isolated by gentle repetitive pipetting and centrifuging of the pipetting cells. (**A**) The scatter plots of PCR Array analysis. At days 0 and 10, total RNA (1 μg) was isolated from HSCs or HSC-derived cells co-cultured with OP9-DLL1/DLL4 cells for the RT^2^ Profiler PCR Array; (**B**) Detection of protein expression of c-Myc and β-actin by immunoblot. Identical results were obtained in three independent experiments.

To explore how the intracellular signals of the Notch pathway induce the differentiation of Ag-specific HSC-T cells, using the RT^2^ PCR Arrays we analyzed the expression of transcription factors in HSCs from OT-I TCR transgenic mice. At day 10 of co-culture, CD3^+^ TCRVβ5^+^ progenitor T cells were sorted. Figure 2A shows that Ag-specific CD3^+^ TCRVβ5^+^ T progenitor cells had a substantially higher c-Myc expression than CD117^+^ HSCs from the bone marrow. As c-Myc is activated by various signals such as Wnt through the MAPK/ERK pathway [13] and c-Myc activation regulates a number of target genes that control many biological effects including proliferation and differentiation, we further examined c-Myc protein expression in CD3^+^ TCRVβ5^+^ T progenitor cells. Like mRNA expression, the overall c-Myc protein expression was higher in CD3^+^ TCRVβ5^+^ T progenitor cells than in CD117^+^ HSCs from the bone marrow. Ligation of Notch with DLL1 and DLL4 resulted in a higher expression of c-Myc on day 10 of the early T progenitor differentiation, and on day 20 of the later T cell maturation (Figure 2B). In light of the importance of c-Myc in cellular activities, c-Myc induction might have a role in regulating HSC-T cell differentiation. This is correlated with the results from c-Myc–deficient mice, which show that such deficiency can lead to defects in the formation of normal lymphocytes [14]. These data suggest that the transcriptional regulation of c-Myc by Notch signals has a role in HSC-T cell differentiation.

### 3.3. c-Myc Directs the Notch Signaling-Mediated Differentiation of HSC-T Cells

**Figure 3:**
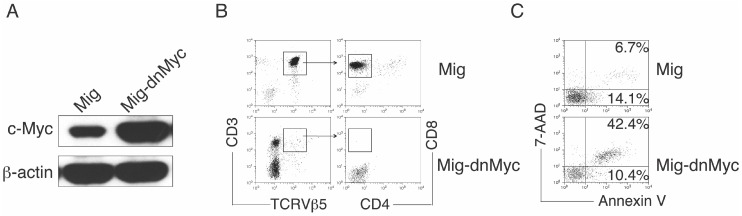
c-Myc directs the Notch signaling–mediated differentiation of HSC-T cells. Bone marrow–derived CD117^+^ HSCs from OT-I TCR transgenic mice were co-cultured on SNL feeders and transduced with retroviral vectors (Mig or Mig–dn c-Myc). Green fluorescent protein (GFP^+^) HSCs cells were isolated and co-cultured with OP9-DLL1/DLL4 cells and cytokines (IL-7 and Flt3L). (**A**) DN c-Myc transduction. GFP^+^ HSCs were isolated and analyzed for c-Myc and β-actin by immunoblot; (**B**) Differentiation of HSC-T cells in vitro. Flow cytometric analysis for the protein expression of HSC-derived cells on day 20; (**C**) Apoptosis of GFP^+^ HSCs on day 4 based on staining of Annexin V and 7-AAD and analyzed by flow cytometry. Data are representative of three independent experiments.

To show the critical role of c-Myc in HSC-T cell differentiation induced by Notch signaling, we transduced CD117^+^ HSCs from the bone marrow with vectors expressing either GFP only (Mig) or GFP plus dn c-Myc (Mig–dn Myc) [10]. GFP^+^ HSCs were sorted, and the amount of c-Myc was examined by immunoblots (Figure 3A). GFP^+^ HSCs were then co-cultured with OP9-DLL1/DLL4 cells and cytokines (IL-7 and Flt3L). Figure 3B shows that gene transduction of DN c-Myc dramatically decreased the differentiation of HSC-T cells, and the percentage of apoptotic cells (Annexin V^+^) was dramatically increased in the group of cells expressing DN c-Myc as compared with those expressing the Mig vector (20.8% vs. 52.8%) (Figure 3C). These observations confirm that transcriptional regulation of c-Myc by Notch signals is essential for HSC-T cell differentiation.

### 3.4. Notch Signaling Affects Survivin Expression during Differentiation of HSC-T Cells

**Figure 4:**
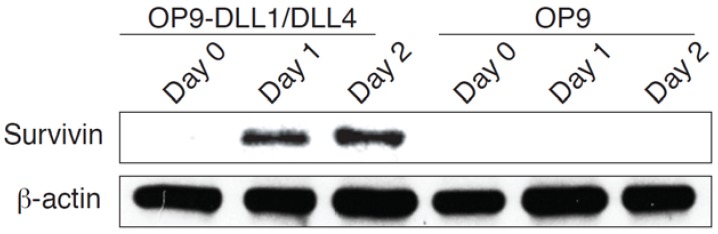
Notch signaling affects survivin expression during HSC-T cell differentiation. The bone marrow-derived CD117^+^ HSCs from OT-I TCR tran sgenic mice were co-cultured with OP9-DLL1/DLL4 or OP9 cells. HSC-derived cells were isolated by gentle repetitive pipetting and centrifuging of the pipetting cells. Expressions of survivin and β-actin were analyzed by immunoblot. Data shown are representative of three identical experiments.

To analyze how HSCs subjected to Notch activation pass the first checkpoint during T cell development, we determined the role of survivin in Notch signaling–mediated HSC-T cell differentiation, as survivin is an important intracellular molecule of Notch signaling and an inhibitor of the apoptosis protein. We stimulated the bone marrow–derived CD117^+^ HSCs on OP9-DLL1/DLL4 cells. At different time points, survivin expression was analyzed by Western blots. We observed that survivin expression in HSCs was up-regulated by 24 h of stimulation, and that induction of survivin required the expression of Notch ligands by the OP9 cells (Figure 4). These results indicate that modulation of survivin expression by Notch signaling also has an important role in the differentiation of HSC-T cells.

### 3.5. c-Myc Controls Survivin Expression in Notch-Activated Cells

**Figure 5:**
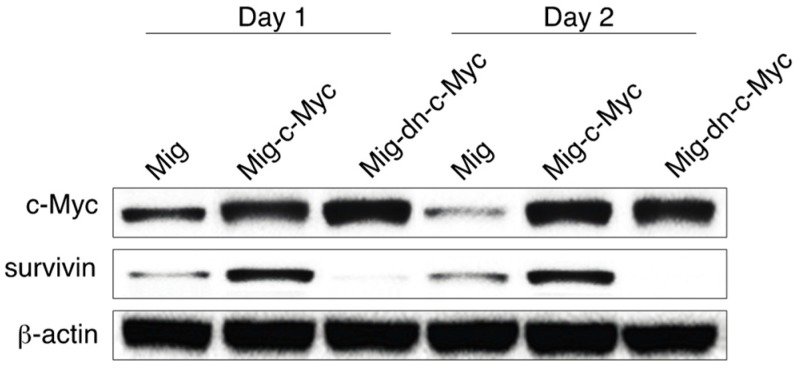
c-Myc controls survivin expression in Notch-activated cells. The bone marrow–derived CD117^+^ HSCs from OT-I TCR transgenic mice were co-cultured on SNL feeders and transduced with retroviral vectors (Mig Mig–c-Myc, or Mig–dn c-Myc). GFP^+^ HSCs cells were sorted and co-cultured with OP9-DLL1/DLL4 cells and cytokines (IL-7 and Flt3L) for two days. GFP^+^ HSCs were sorted and analyzed for survivin and β-actin by immunoblot. Data shown are representative of three identical experiments.

c-Myc facilitates many cellular activities of T cells, including proliferation, differentiation, homeostasis and metabolic reprogramming. We previously showed that sustained survivin expression from T cell costimulation regulates cell expansion and memory [10,12]. Hence, we explored if c-Myc mediates HSC-T cell differentiation through survivin. We found that gene transduction of c-Myc in the bone marrow–derived CD117^+^ HSCs up-regulated survivin expression, whereas DN c-Myc substantially reduced survivin expression (Figure 5). These results are similar to the reported effects in different transformed cells in which c-Myc also mediated survivin expression [15,16]. These data indicate that survivin is needed for the transcriptional regulation of HSC-T cell differentiation by c-Myc in the Notch-activated cells.

### 3.6. Survivin-Deficient HSCs Show Reduced Ability to Differentiate into T Cells in Response to Notch Signaling

**Figure 6:**
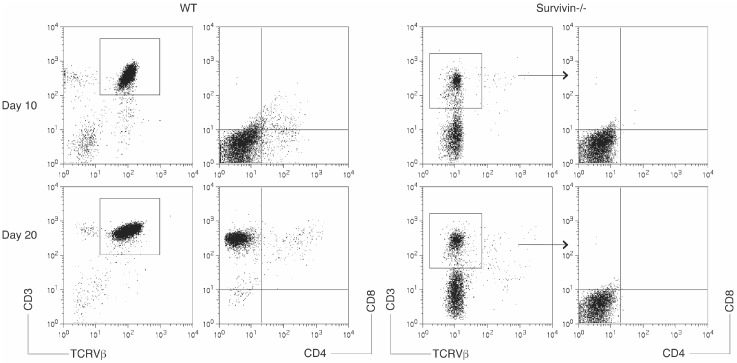
Survivin-deficient HSCs show reduced ability to differentiate into T cells in response to Notch signaling. The bone marrow–derived CD117^+^ HSCs from the wild type or survivin^−/−^ mice were co-cultured with the OP9-DLL1/DLL4 cells and cytokines (IL-7 and Flt3L). Differentiation of HSC-T cells was examined by flow cytometric analysis for the protein expression in the HSC-derived cells on days 10 and 20. CD3^+^ TCRVβ^+^ cells were gated as indicated, and analyzed for the expression of CD4 and CD8. Data shown are representative of three identical experiments.

Survivin has emerged as an essential mediator of T cell expansion, coupling cell cycle progression to apoptosis resistance [12]. To determine the functional role of survivin in the differentiation of HSC-T cells, we utilized an in vitro differentiation system as described in Figure 1. The bone marrow–derived CD117^+^ HSCs from the survivin-conditional deficient mice (survivin^−/−^ mice; under control of the lck promoter) [17] were stimulated with the OP9-DLL1/DLL4 cells and cytokines (rIL-7 and Flt3L). The selective expression of the TCR Vβ chain but not CD3 was inhibited, and a four-week co-culture did not generate OVA-specific CD3^+^TCRVβ^+^ T cells from survivin^−/−^ HSCs (Figure 6). These results suggest that survivin promotes the differentiation of HSC-T cells driven by Notch signaling.

## 4. Discussion

The Notch system controls the fate of HSCs in the thymus as they progress along the differentiation processes. Although Notch1 receptor (N1R) is the key Notch receptor involved in T cell lineage commitment and thymic T cell maturation, the ligands that can be the biological companions of N1R in these developments are still not clear. In addition, the interaction between Notch3 receptor (N3R) or 4 receptor (N4R) with Notch ligands is also associated with T cell development. Several groups have shown that Notch signaling is indispensable in inducing T cell differentiation from stem cells [18,19,20,21], and we recently showed that Notch signaling promotes T cell differentiation from stem cells [9,22,23,24]. Here, we generated a new OP9 stromal cell line (i.e., OP9-DLL1/DLL4) expressing DLL1 and DLL4 molecules, and this cell line induces HSC-T cell differentiation in vitro.

Little is known about how Notch signaling induces HSCs to pass the first checkpoint during T cell development. Survivin is a crucial molecule that affects numerous cellular processes such as apoptosis, proliferation, differentiation, and metastasis. Survivin homozygous knockout mice prematurely die at embryonic day 3.5 with defects of cell proliferation, spindle formation, and apoptosis, demonstrating the requirement of survivin during cell development [25]. Knockdown of survivin induces dual phenotypes of mitotic defects (e.g., centrosomal abberations, multipolar spindles, and chromatin missegregation) as well as apoptosis in T cells [17,26]. We have shown that sustained survivin expression promotes T cell clonal expansion [12,27]. In addition, we have reported that survivin as a serine-threonine kinase physically associates with aurora B and mammalian target of rapamycin (mTOR) to regulate the G1-S cell cycle progression of T cells [28]. In this study, we demonstrate that Notch signaling acts through the regulation of survivin to control β-selection during thymocyte differentiation.

The transcriptional regulation of Notch signaling plays a central role in cell proliferation, differentiation and function, and a number of transcriptional factors have been identified, including Nfkb2, Id1 and Stat6. Using the transcription factor PCR array, we demonstrated that c-Myc is also a crucial intracellular molecule of Notch signaling which enhances the differentiation of HSC-T cells. c-Myc is a proto-oncogene which adjusts abundant cellular activities, such as proliferation, growth, self-renewal, and differentiation, in addition to apoptosis [29,30,31]. c-Myc is needed throughout cell and tissue development, as c-Myc deficiency can cause primary embryonic lethality, and c-Myc dysfunction in hematopoietic cells drives the defeat of the main myeloid and lymphoid lineages [32,33]. A transgenic c-Myc expression in various tissues induces neoplastic diseases [34], demonstrating the c-Myc vigor being an oncogene. c-Myc is essential for T cell development; however, previous results suggest that the role of c-Myc in the regulation of T cell development is in proliferation. c-Myc was identified to be an important direct target of Notch1 in T cell acute lymphoblastic leukemia/lymphoma (T-ALL), in which c-Myc inhibitors prevented Notch1 from rescuing T-ALL cells treated with γ-secretase inhibitor (GSI), and c-Myc overexpression rescued T-ALL cell lines from GSI-induced growth arrest [35,36,37]. Furthermore, Notch1 may control c-Myc enhancer to promote T cell development, transformation and T-ALL [38]. These results suggest that c-Myc highly promotes cell proliferation and survival. Importantly, the role for c-Myc in T lymphocyte differentiation from HSCs induced by Notch signaling has not been previously determined. We recently showed that c-Myc regulation from costimulatory signaling controls memory T cell development [10]. Using an in vitro T cell differentiation system, we now show that c-Myc is a critical intracellular target of Notch signaling that promotes the differentiation of HSC-T cells.

**Figure 7:**
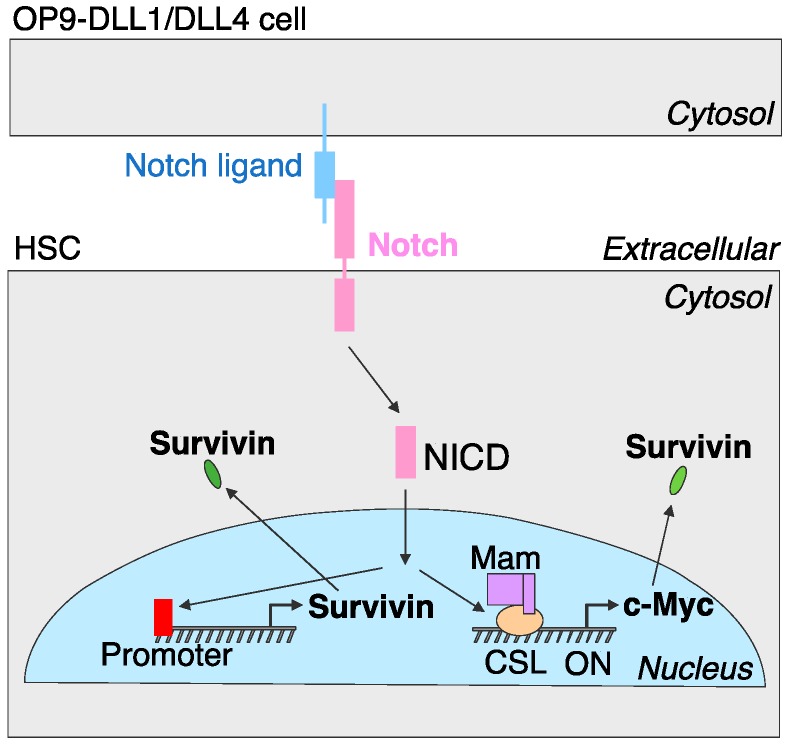
Model for Notch–c-Myc–survivin axis. The interaction between the Notch ligand and Notch receptor results in the transcriptionally active NICD, which translocates to the nucleus. In the nucleus, the NICD (i) interacts with CSL and Mam proteins, and induces transcription of c-Myc which subsequently regulates the expression of survivin involved in Notch-dependent T cell differentiation, and/or (ii) directly activates survivin by recognition of the survivin promoter region.

The results reported here indicate that the Notch–c-Myc–survivin axis is indispensable for T cell differentiation. The ligation of the Notch receptor with a ligand by the canonical Notch signaling pathway induces endocytosis and membrane trafficking, generation of the membrane-anchored Notch extracellular truncation fragment (a substrate for γ-secretase complex) as well as the cleavage of the NICD. NICD then translocates to the nucleus where it links with the DNA-binding protein CSL (CBF1/RBPjκ/Su(H)/Lag-1). The transcriptional coactivator Mastermind (Mam) recognizes the NICD/CSL interface, and this three-protein complex causes coactivators to activate transcription [4]. It is possible that Notch1 directly activates survivin, and a canonical Notch1 recognition sequence may be proximal to the survivin promoter region (Figure 7). Further studies are needed to identify this possibility.

## 5. Conclusions

The current study demonstrates the critical function of the Notch–c-Myc–survivin axis in HSC-T cell differentiation. We show that Notch signaling induces c-Myc to enhance the downstream target survivin, which promotes the differentiation of HSC-T cells. Other downstream targets might also be involved in HSC-T cell differentiation via Notch signaling and their functions remain to be elucidated.
